# Supplementary figures and images for: Mutational impact of APOBEC3A and APOBEC3B in a human cell line and comparisons to breast cancer
Source: PLoS Genet. 2023 Nov 30;19(11):e1011043. doi: 10.1371/journal.pgen.1011043 (PMC10715669; doi:10.1371/journal.pgen.1011043)

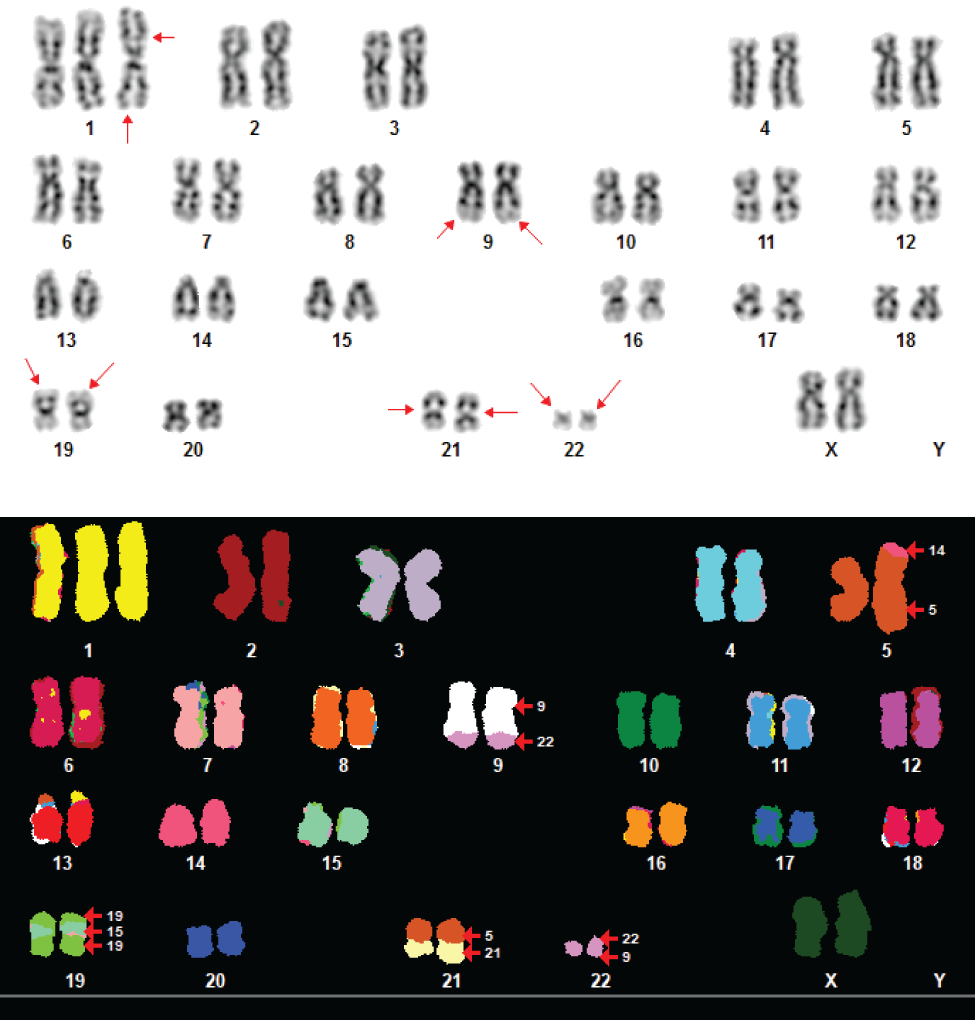

Supplement: S1 Fig — Representative G-band and spectral karyotype (SKY) images of HAP1-TK-M9 M-phase chromosomes showing a near diploid DNA content and previously reported aberrations including the reciprocal chromosome 9:22 translocation (Philadelphia chromosome) characteristic of CML tumor cells. The Y-chromosome is missing, as reported for the KBM7 parent line of HAP1. (TIF) [file pgen.1011043.s003.tif]

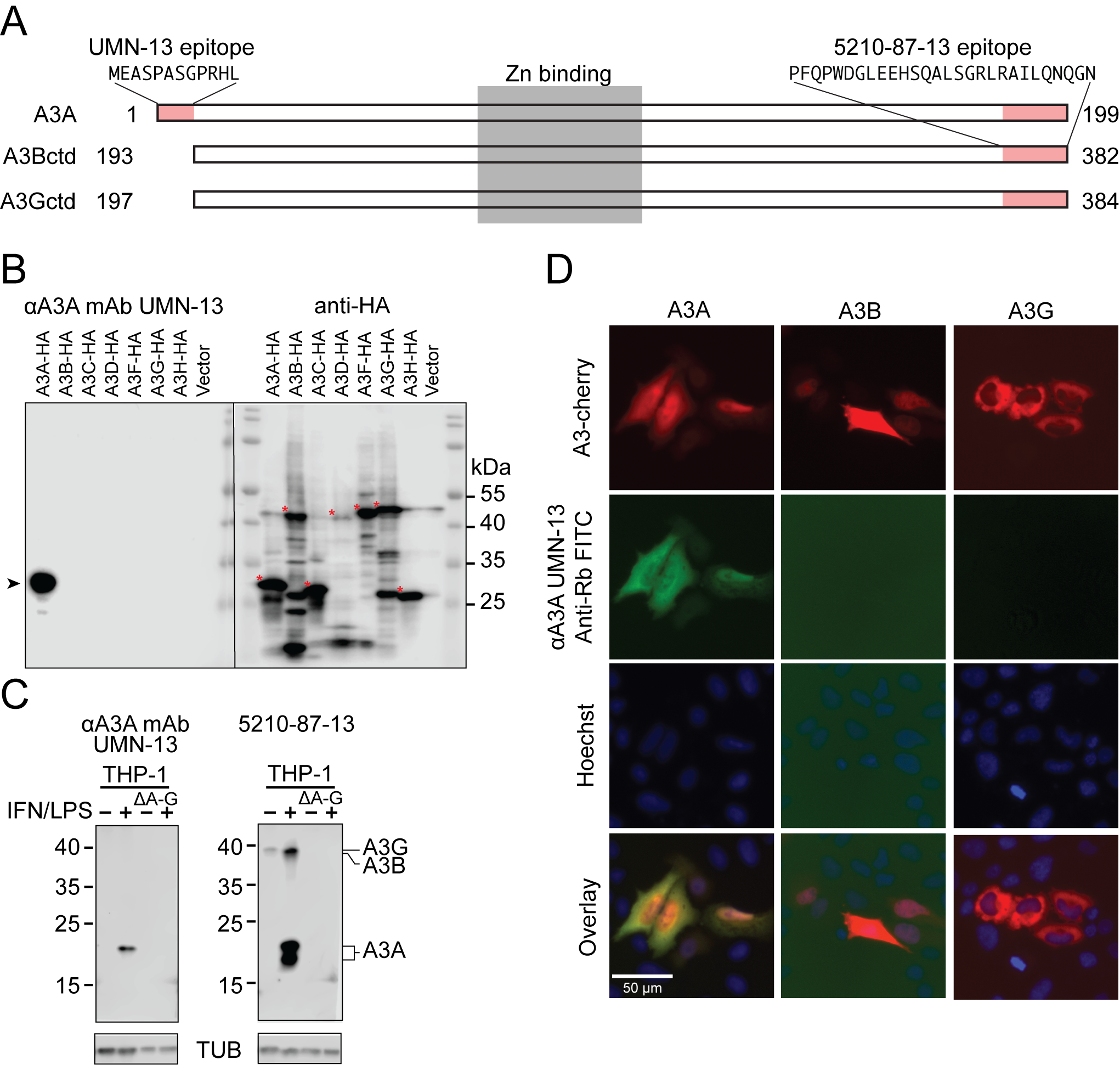

Supplement: S2 Fig — (A) Schematic of human A3A, A3B, and A3G indicating the unique N-terminal epitope used here to generate the A3A-specific mAb UMN-13. The schematic also shows the C-terminal epitope used previously to generate the versatile 5210-87-13 mAb that recognizes these three enzymes. (B) Comparative immunoblots of whole cell extracts from 293T cells expressing each of the 7 human A3 family members with C-terminal HA tags. The blot was probed first with our custom rabbit anti-human A3A mAb UMN-13 (left) and, after stripping, a commercial anti-HA mAb as an expression control (right). The positions of the full-length proteins are indicated by red asterisks. (C) Comparative immunoblots of whole cell extracts from the monocytic cell line THP-1 and a clonal derivative lacking A3A-through-A3G (ΔA-G), each treated with DMSO as a control or LPS/IFN-α to induce expression of multiple A3s including A3A and A3G. The UMN-13 mAb blot on the left shows a single band representing full-length A3A (starting at Met1), which is absent in the deletion mutant, and the 5210-87-13 mAb blot on the right shows A3G (strong top band), A3B (weak band just below A3G), and both A3A translation products (strong band for full-length A3A starting at Met1 and a faster-migrating band for the shorter isoform starting at Met13), which are all absent in the deletion mutant. (D) IF microscopy images of 293T cells expressing A3A-mCherry, A3B-mCherry, or A3G-mCherry. Only the A3A construct is detected by the UMN-13 mAb as indicated by green signal in the same cells and cellular compartments as the A3A-mCherry signal. (TIF) [file pgen.1011043.s004.tif]

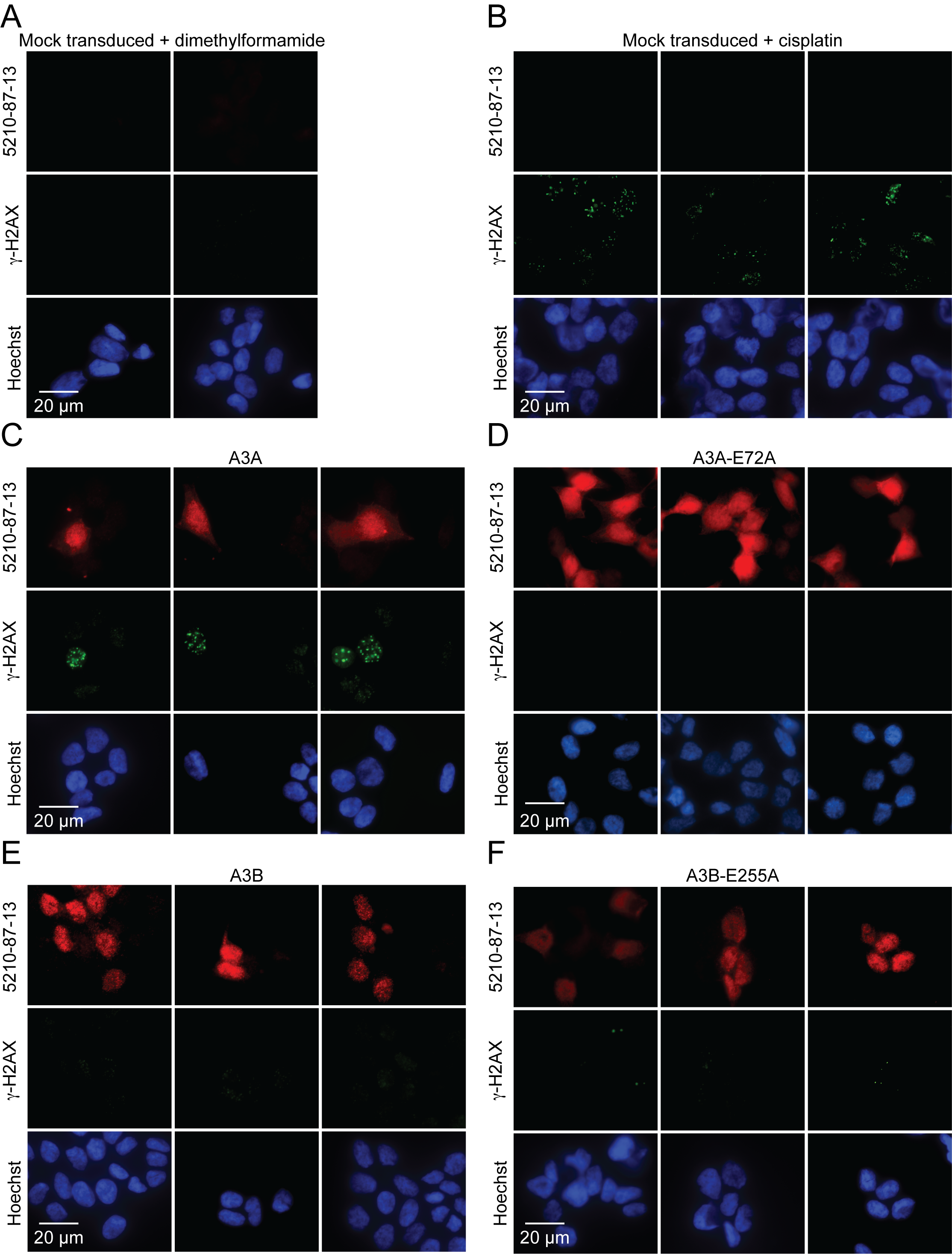

Supplement: S3 Fig — (A-B) Additional IF-images of HAP1-TK-M9 cells mock-transduced and DMF or cisplatin treated, respectively, and stained as indicated in parallel with cells in panels C-F (scale = 20 μm). (C-F) Additional IF-images of HAP1-TK-M9 cells transduced with the indicated A3 expression constructs and stained with the 5210-87-13 rabbit anti-human A3A/B mAb, γ-H2AX, and Hoechst (scale = 20 μm). (TIF) [file pgen.1011043.s005.tif]

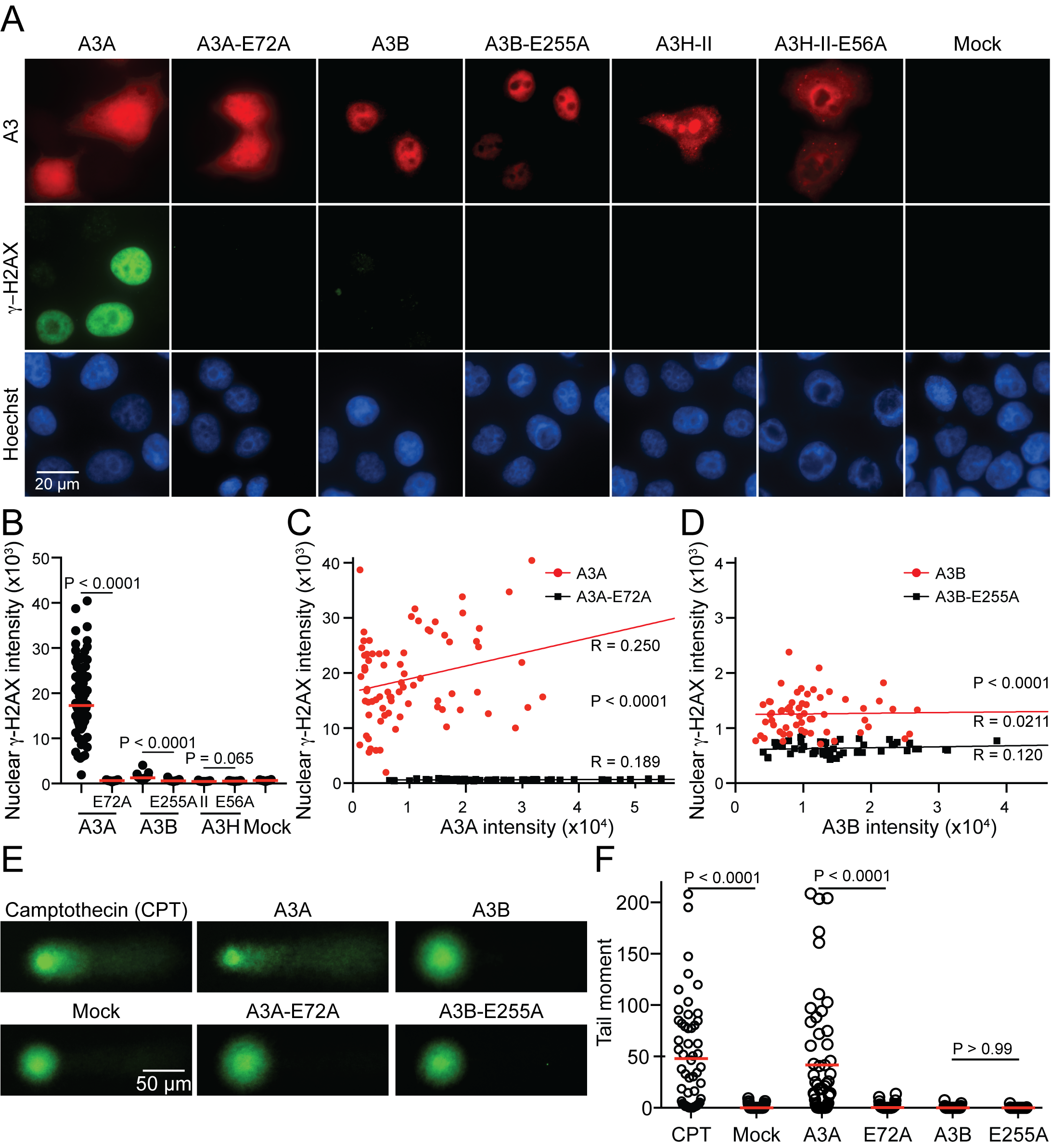

Supplement: S4 Fig — (A) IF-images of HeLa cells transfected with the indicated A3 expression constructs and stained for A3A/B (5210-87-13 rabbit anti-human A3A/B mAb) or A3H (Novus ARP10 rabbit anti-human A3H pAb), γ-H2AX, and Hoechst (scale = 20 μm). (B) Quantification of nuclear γ-H2AX staining intensity in the different A3 expressing conditions in panel A (n>50 cells per condition; red bars indicate mean expression levels; p-values by Welch’s t-test). (C) Dot plot of nuclear γ-H2AX staining intensity versus A3A staining levels for A3A and A3A-E72A expressing cells shown in panel B. Correlation coefficients (R-values) indicate that A3A expression levels fail to correlate with nuclear γ-H2AX staining intensity. The p-value by Welch’s t-test reflects the difference between that A3A and A3A-E72A data sets. (D) Dot plot of nuclear γ-H2AX staining intensity versus A3B staining levels for A3B and A3B-E255A expressing cells shown in panel B. Correlation coefficients (R-values) indicate that A3B but not A3B-E255A expression levels associate with nuclear γ-H2AX staining intensity. The p-value by Welch’s t-test reflects the difference between that A3B and A3B-E255A data sets. (E) Representative comets from HeLa cells mock transduced, treated with 2 μM camptothecin (CPT), or transduced with expression constructs for A3A, A3B, or catalytic mutant derivatives (scale = 50 μm). (F) Quantification of tail moment for >50 cells per condition indicated in panel E (red bars indicate mean tail moment; p-values by one-way ANOVA). (TIF) [file pgen.1011043.s006.tif]

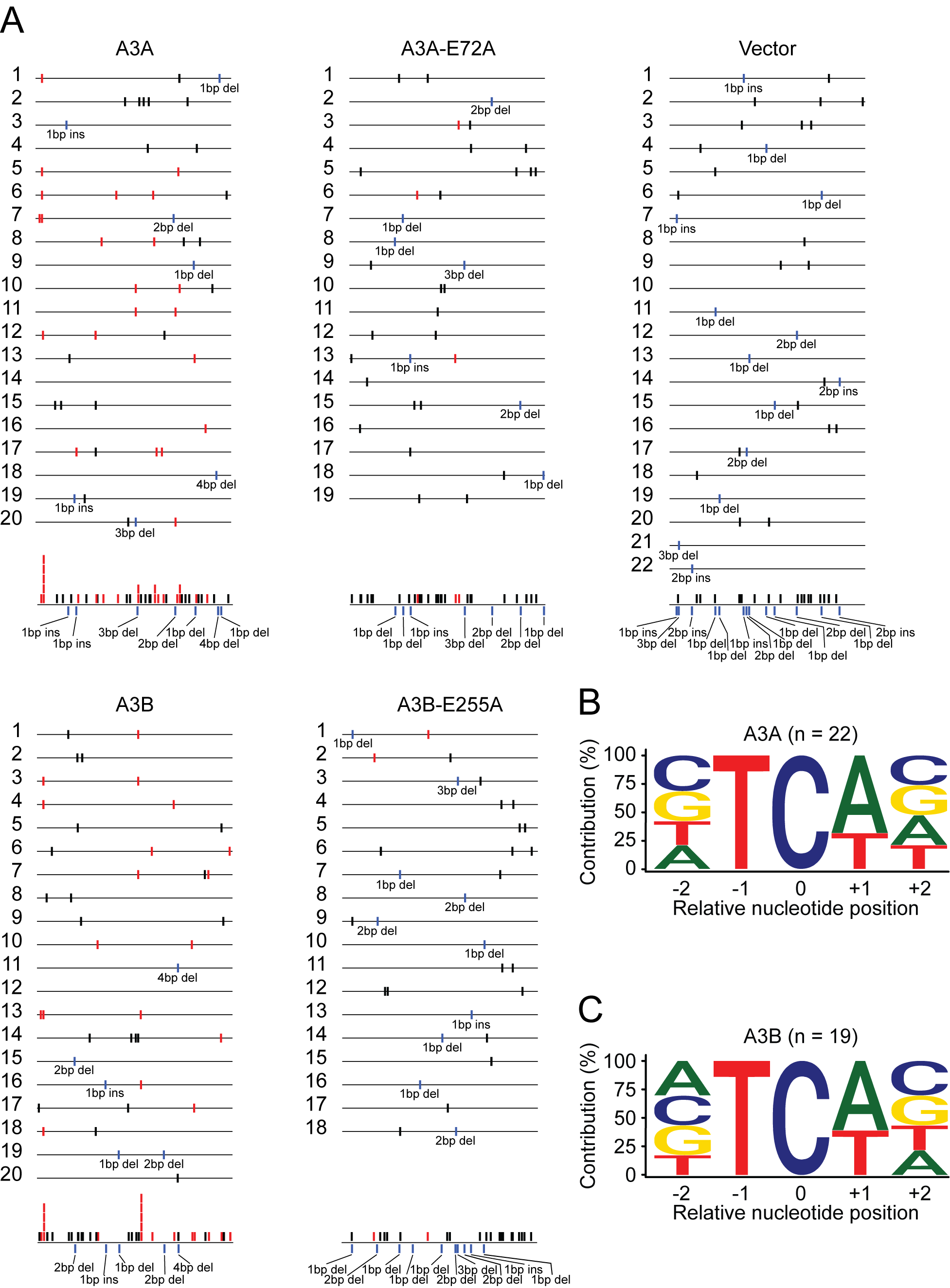

Supplement: S5 Fig — (A) Schematics of individual TK mutations in ganciclovir-resistant clones. T[C>G/T]W mutations are shown in red, other SBSs in black, and INDELs in blue. Composite mutation schematics are shown below for each condition. (B-C) Pentanucleotide logos depicting the -2 and +2 sequence preferences flanking all T(C>T/G)W mutations that accumulated in TK during expression of the indicated constructs. (TIF) [file pgen.1011043.s007.tif]

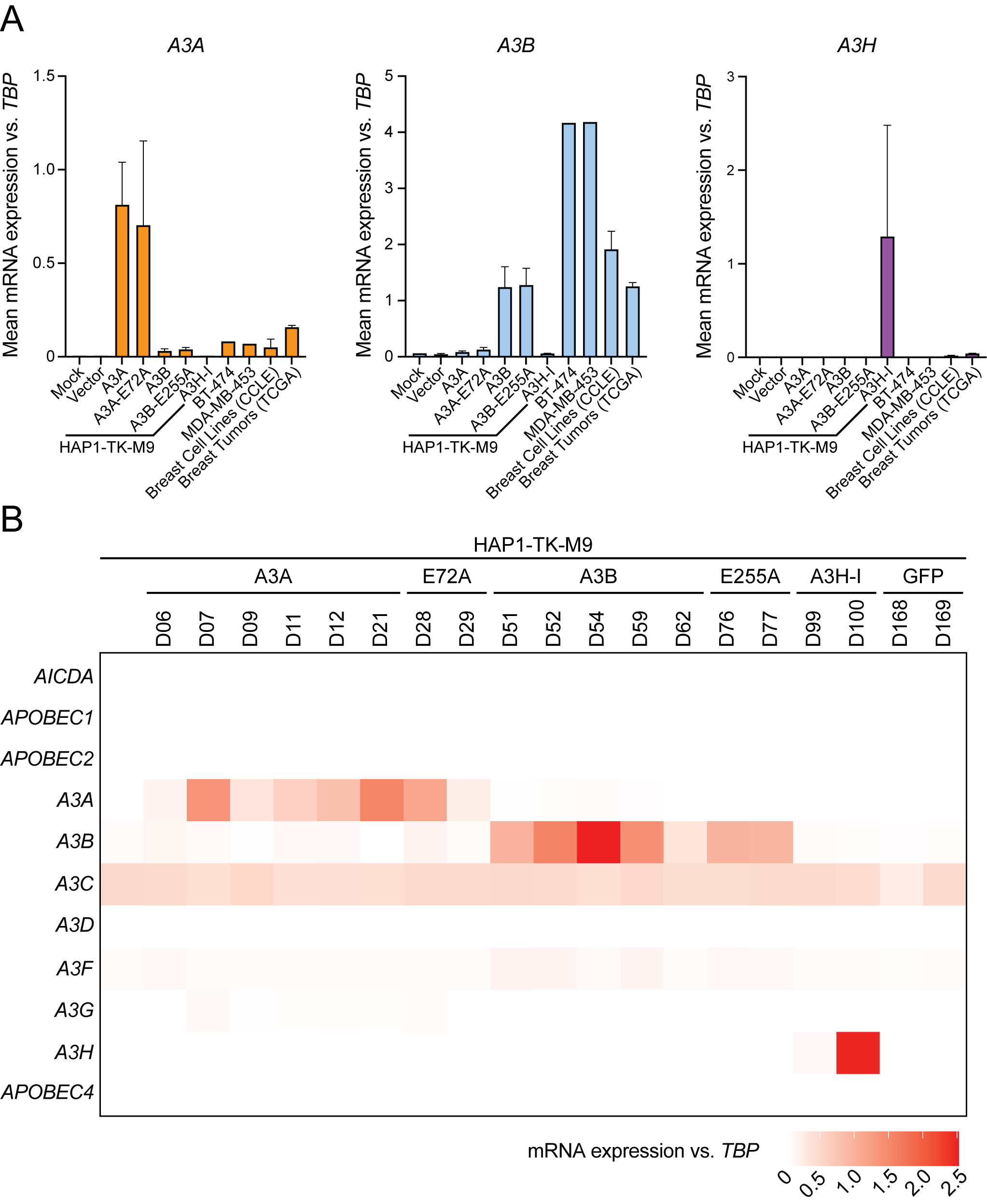

Supplement: S6 Fig — (A) A3A, A3B, and A3H mRNA expression levels relative to those of the housekeeping gene TBP for the indicated HAP1-TK-M9 conditions (RNA-seq FKPM from n≥2 clones for each condition; mean +/- SD shown). RNA-seq data from A3 signature-high breast cancer cell lines (BT-474 and MDA-MB-453), CCLE breast-derived cell lines (n = 52), and TGCA primary breast cancers (n = 1093) for comparison (mean +/- SD). (B) A heatmap depicting mean expression levels of all 7 human APOBEC3 family members, in addition to AICDA, APOBEC1, APOBEC2, and APOBEC4, relative to those of the housekeeping gene TBP (RNA-seq values are FKPM; n≥2 for each condition to provide matching data sets for clones subjected to WGS). Endogenous A3C provides a consistent internal control. (TIF) [file pgen.1011043.s008.tif]

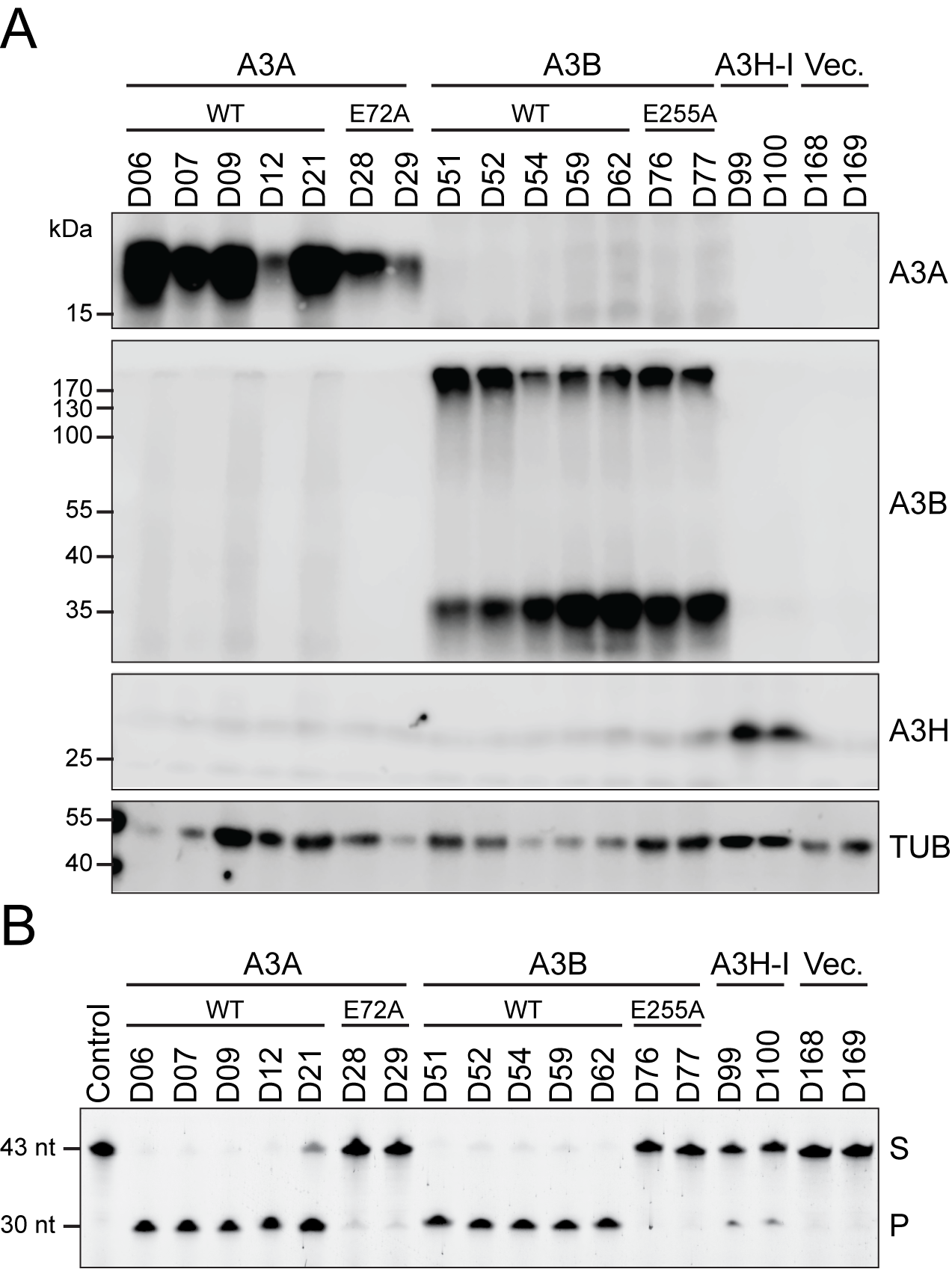

Supplement: S7 Fig — (A) Immunoblots of A3A, A3B, and A3H in the indicated clones. Tubulin (TUB) is a loading control. (B) Deaminase activity of WCE on ssDNA from the same clones (S, substrate; P, product). (TIF) [file pgen.1011043.s009.tif]

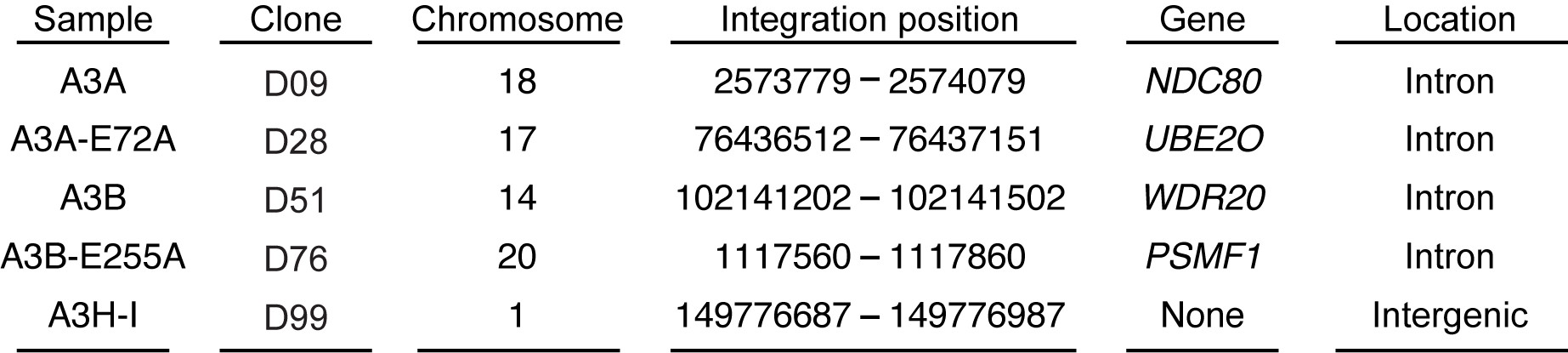

Supplement: S8 Fig — A table indicating chromosomal locations of representative MLV-A3 insertion sites in HAP-1-TK-M9 clones. In each clone, a single MLV-A3 insertion is positioned in the window between the indicated nucleotides. (TIF) [file pgen.1011043.s010.tif]

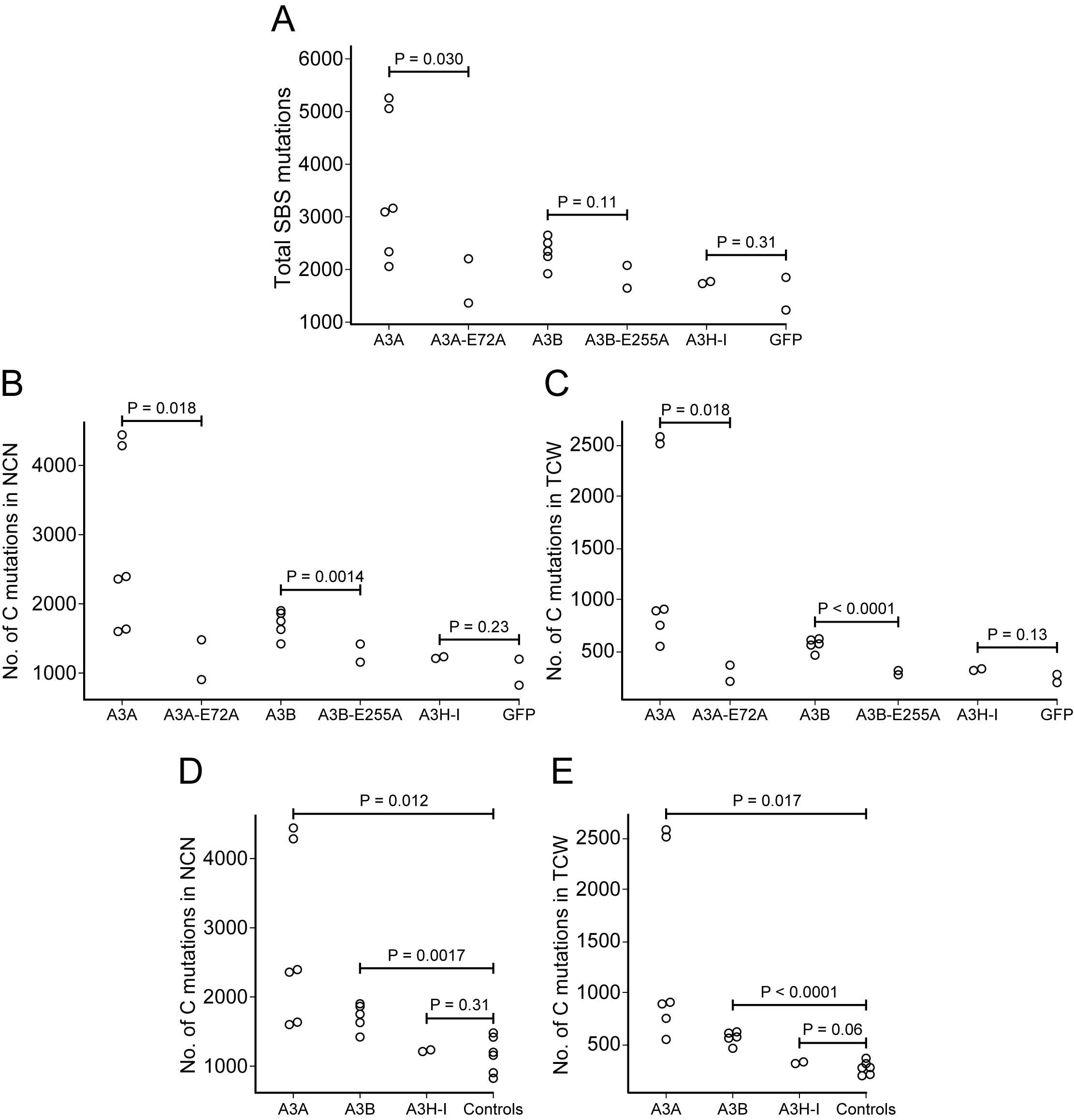

Supplement: S9 Fig — (A-C) Dot plots showing total numbers of SBS mutations and cytosine mutations in NCN and TCW motifs, respectively, in WGS from individual granddaughter clones (p-values using Welch’s t-test). (D-E) Dot plots showing total numbers of cytosine mutations in NCN and TCW motifs, respectively, in WGS from individual A3A and A3B expressing granddaughter clones in comparison to all non-catalytic controls combined (p-values using Welch’s t-test). (TIF) [file pgen.1011043.s011.tif]

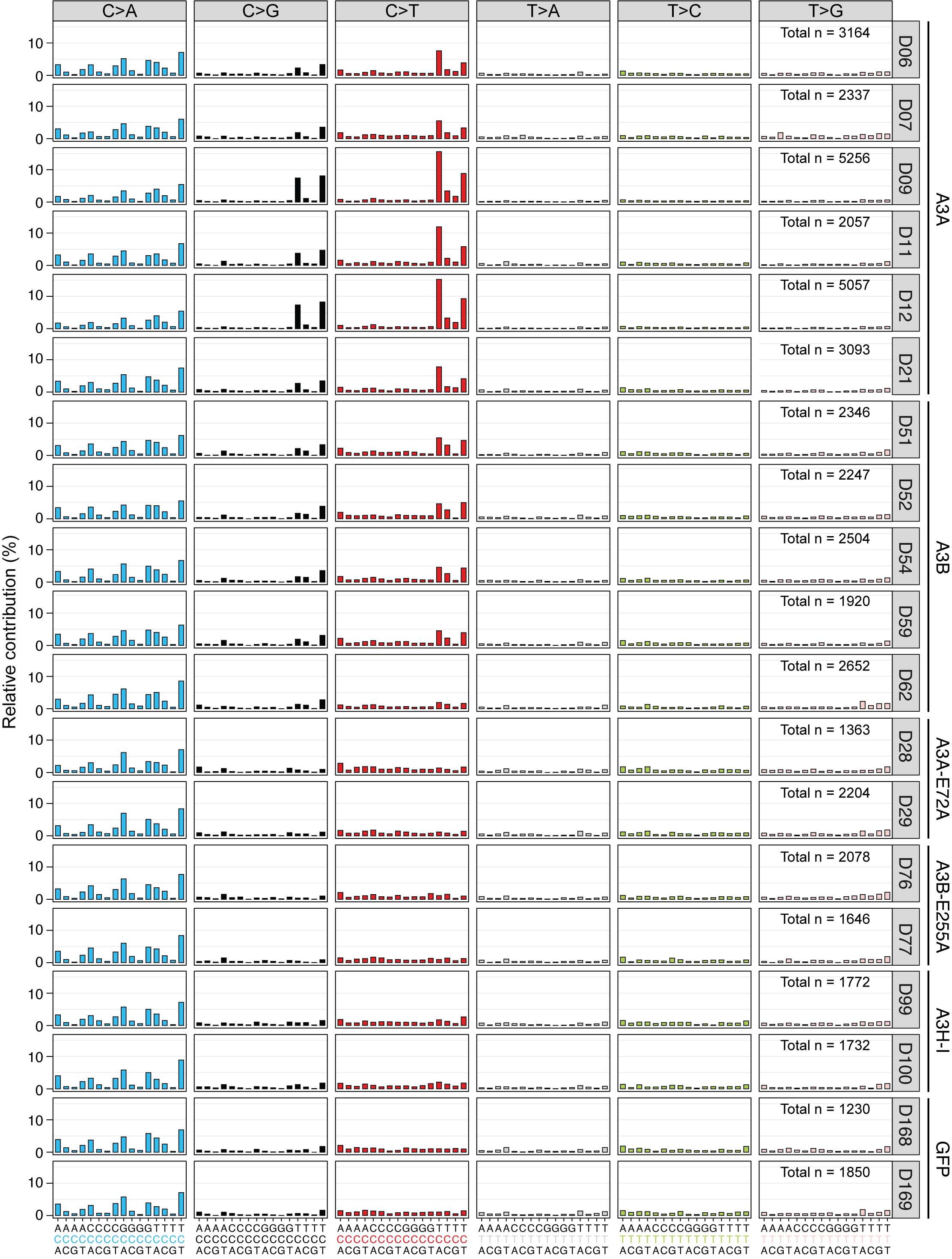

Supplement: S10 Fig — Trinucleotide profiles of all SBS mutations in WGSs from the indicated granddaughter clones (conditions, clone names, and total SBS numbers are indicated to the right in each profile). Aggregate profiles for A3A, A3B, and corresponding catalytic mutant controls are shown in Fig 3. (TIF) [file pgen.1011043.s012.tif]

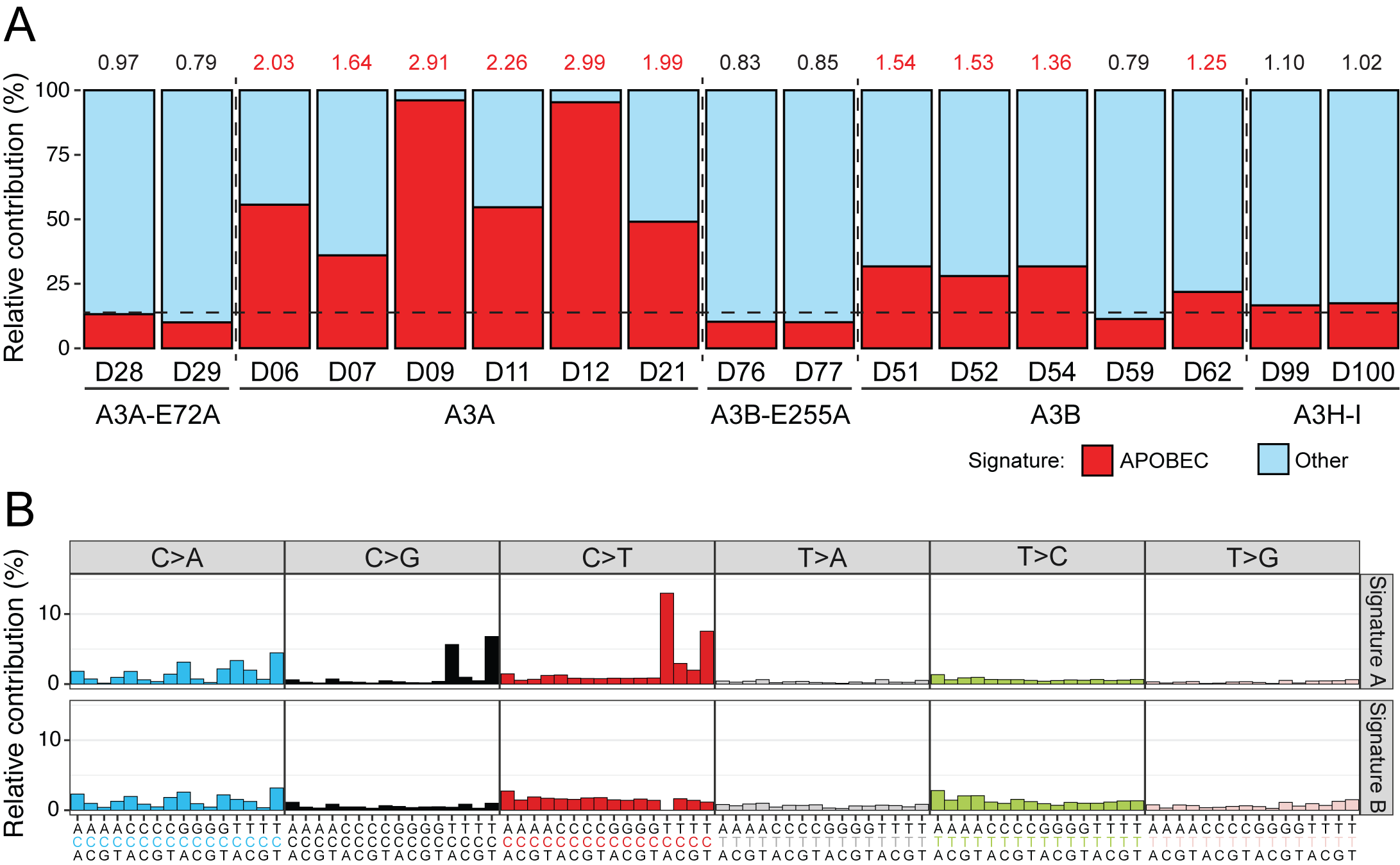

Supplement: S11 Fig — (A) Mutation signature profiles extracted from granddaughter clone WGS using an NMF-based approach. The SBSs of each clone resolved into 2 signatures—an APOBEC3-like mutation signature shown in red and a background signature in blue. APOBEC3 mutation enrichment scores are shown above each bar, with significantly enriched values shown in red (Benjamini-Hochberg false discovery rate-corrected q-value < 0.05). The dashed line represents the average level of APOBEC3 signature mutations observed in the two eGFP control clones (i.e., background signal). (B) Trinucleotide mutation profiles of signatures A and B derived using NMF with the former exhibiting an APOBEC3 SBS signature (C-to-T and C-to-G in TCA and TCT motifs). (TIF) [file pgen.1011043.s013.tif]

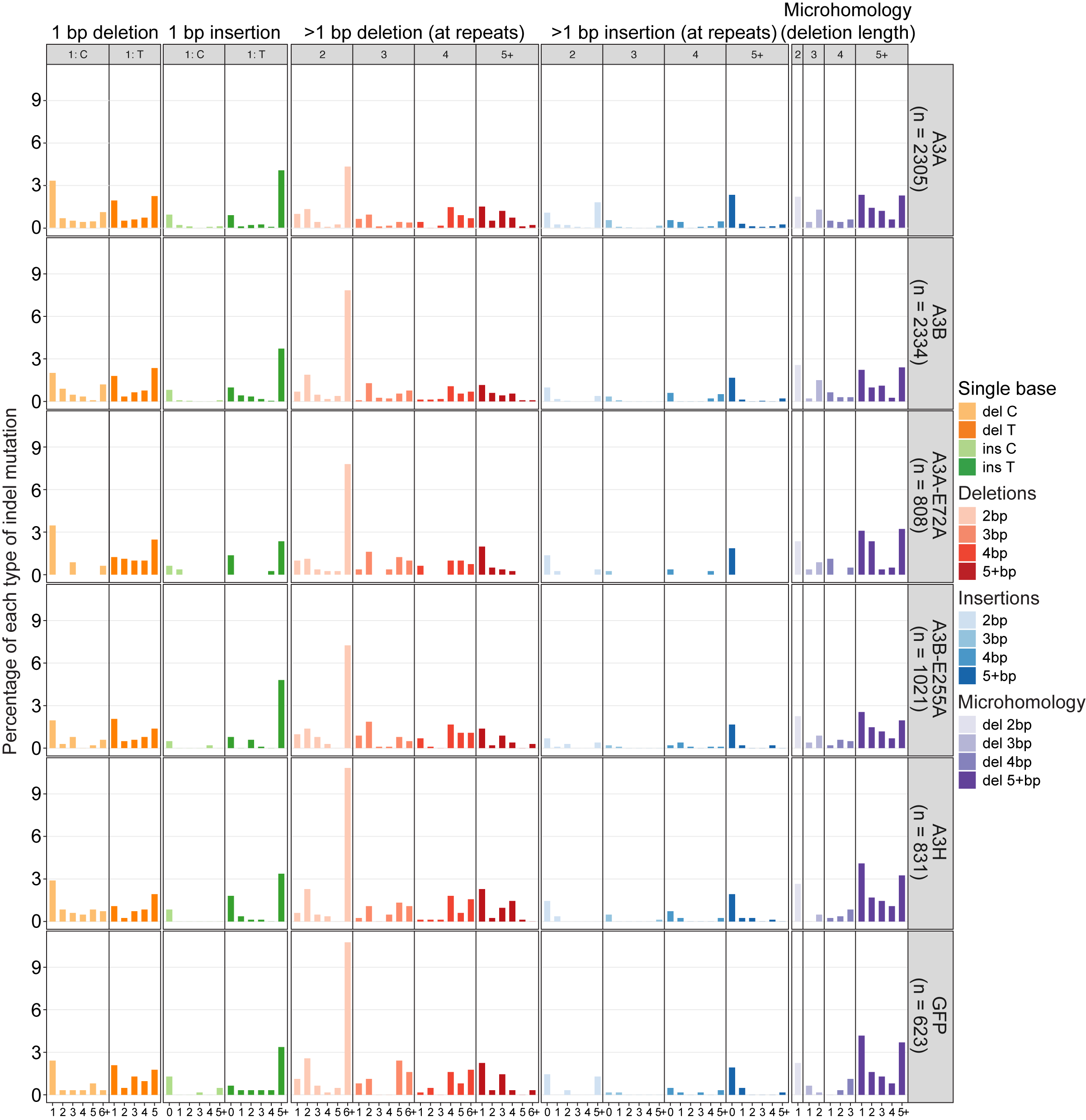

Supplement: S12 Fig — Bar plots showing the percentage of each of the indicated indel types occurring in WGS from HAP1-TK-M9 granddaughter clones. Total numbers of single T deletions at homopolymers of 6 or more are too numerous to plot on the same axis and are therefore listed here (A3A, 39.5%; A3B, 44.2%; A3A-E72A, 40.6%; A3B-E255A, 40.0%; A3H, 31.8%; and eGFP, 27.4%). The cosine similarity of the indel landscape across all conditions is over 0.96 indicating no significant differences. (TIF) [file pgen.1011043.s014.tif]

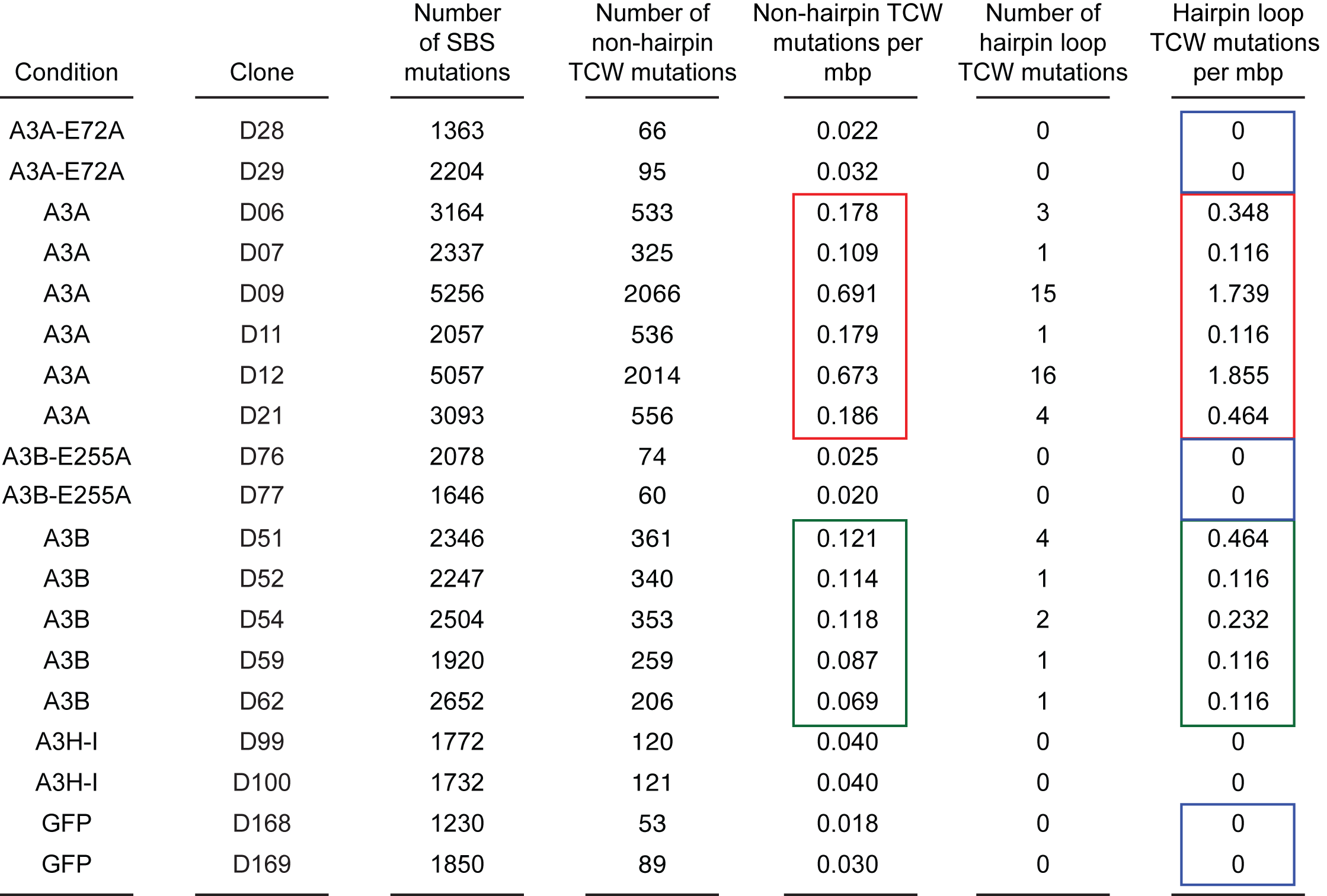

Supplement: S13 Fig — A list of the total number of SBS mutations in each HAP1-TK-M9 clone and numbers and frequencies mutations predicted to occur in non-hairpin regions or hairpin loop regions of the genome (see Methods for additional information). Columns 1 and 2: The A3 or control condition and clone number as listed in S5 Fig. Column 3: The total number of SBS mutations in each clone by WGS (human genome: 3000 mbp). Columns 4 and 5: The total number and frequency of non-hairpin APOBEC3 signature TCW mutations per clone (estimated non-hairpin genomic DNA: 2991.375 mbp). Columns 6 and 7: Total number and frequency of APOBEC3 signature TCW mutations in 3–11 nucleotide loop regions of predicted chromosomal DNA hairpin structures (estimated ssDNA loop region genomic DNA: 8.625 mbp). For A3A data (red boxes) and A3B data (green boxes), the non-hairpin versus hairpin APOBEC3 TCW mutation frequencies are not significantly different (P = 0.25 and P = 0.19, respectively, by Welch’s t-test). APOBEC3 TCW mutation frequencies are also not significantly different between the A3A and A3B data sets (red versus green boxes) for both non-hairpin regions (P = 0.087) or predicted ssDNA loop regions of hairpins (P = 0.149). However, in comparisons of A3A (red) and A3B (blue) APOBEC3 TCW mutation frequencies in ssDNA loop regions of hairpins and equivalent data sets from aggregate controls (blue boxes: the catalytic mutant of each protein and eGFP), the A3A data set approaches statistical significance (P = 0.0655) and the A3B data are significantly different (P = 0.0367) by Welch’s t-test. (TIF) [file pgen.1011043.s015.tif]

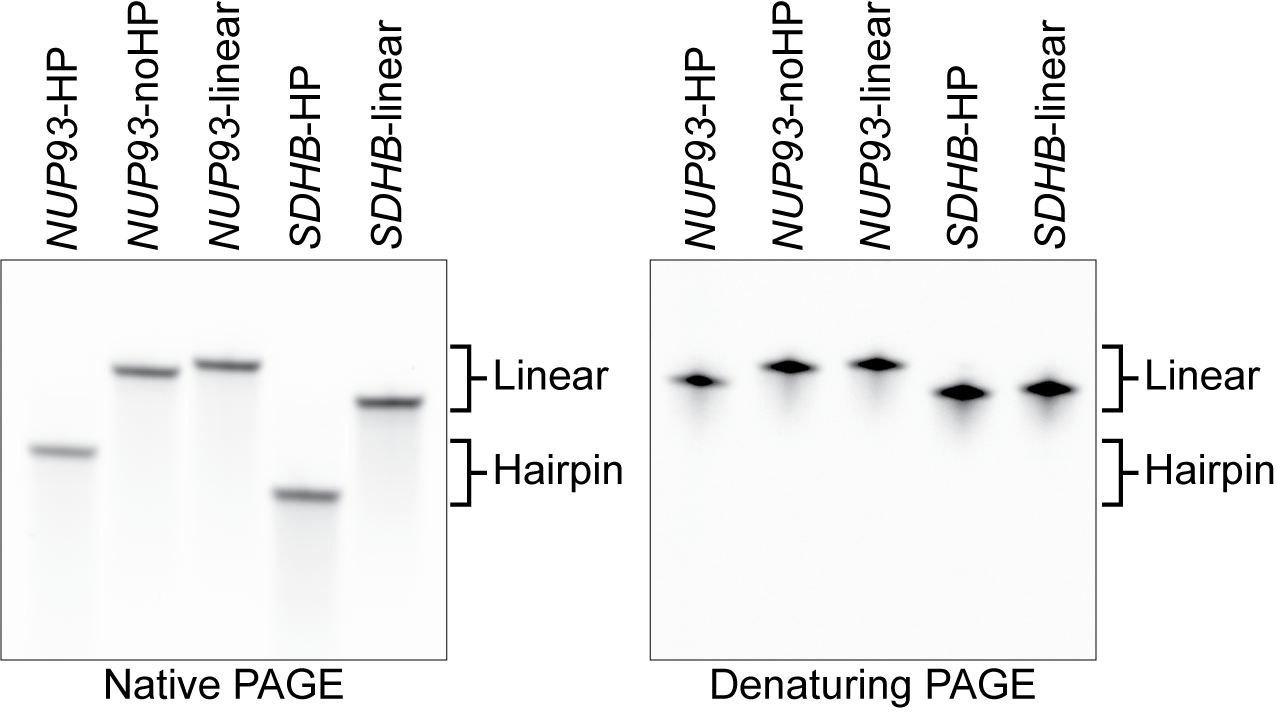

Supplement: S14 Fig — Native PAGE (left) and denaturing PAGE (right) analysis of the indicated oligonucleotide substrates. The hairpin substrates migrate faster under native conditions, and their mobility is similar to the linear derivatives under denaturing conditions. The only oligonucleotide not used in biochemical experiments in Fig 4 is the NUP93-noHP (no hairpin), which has half of the stem replaced by adenines (5’-6-carboxyfluorescein-GCAAGCTGTTCAAAAAAATGA) and is included here as an additional control. (TIF) [file pgen.1011043.s016.tif]

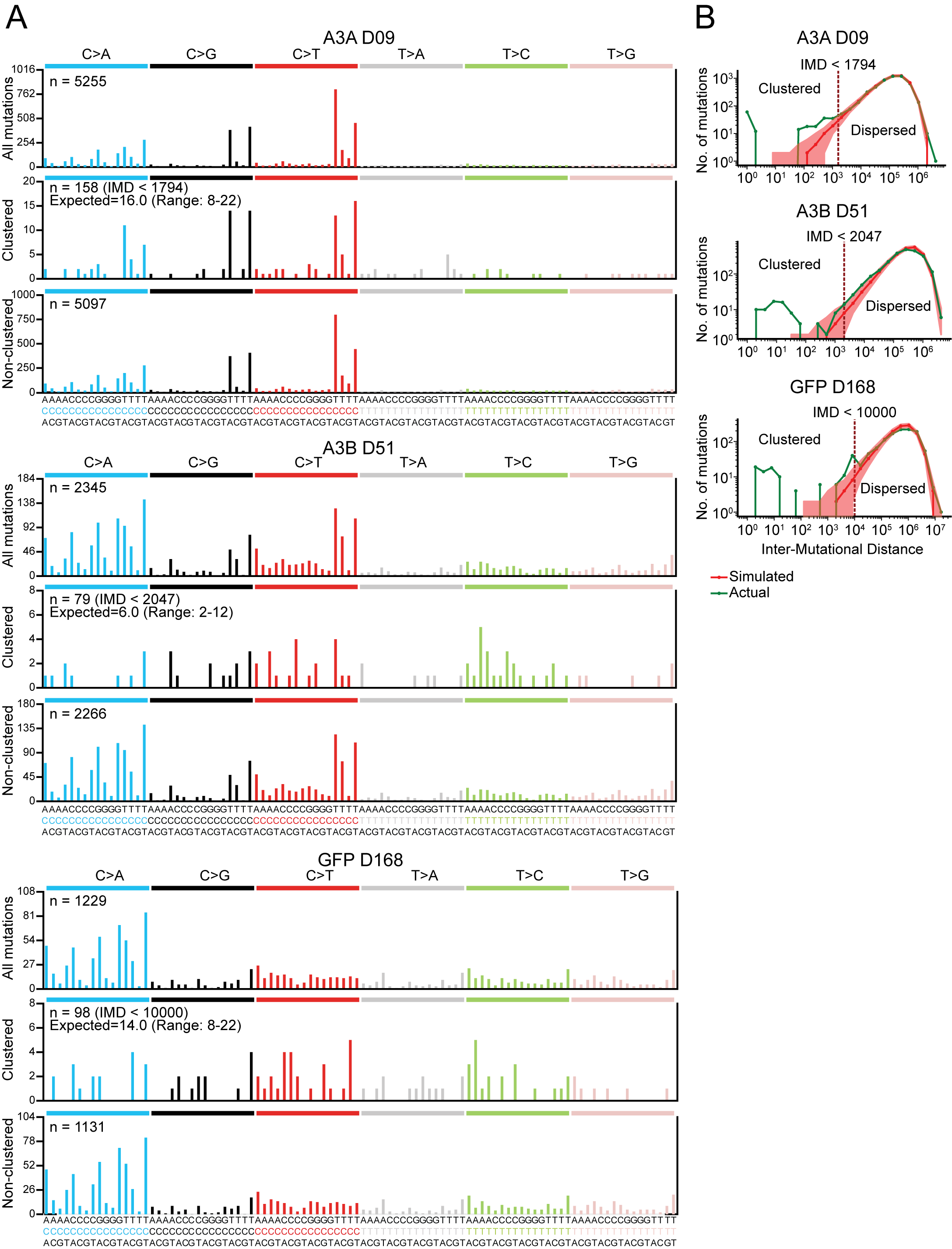

Supplement: S15 Fig — (A) Trinucleotide profiles of total, clustered, non-clustered single base substitution substitution mutations in representative A3A (top), A3B (middle), and eGFP (bottom) clones. The intermutation distance (IMD) is indicated, together with the expected number number of kataegis events and the actual range of kataegis events observed for clones of each condition determined using SigProfilerClusters (Materials and Methods). (B) IMDs of A3A (top), A3B (middle), and eGFP (bottom) clones. Green lines are based on actual intermutation distances, and red lines are simulated distributions of intermutation distances. 95% confidence intervals are shown in pink for the simulated IMD distributions. (TIF) [file pgen.1011043.s017.tif]
